# Isolation and identification of EST‐SSR markers in *Ixonanthes chinensis* (Ixonanthaceae)

**DOI:** 10.1002/aps3.1246

**Published:** 2019-05-10

**Authors:** Wei Guo, Qiang Fan, Jinjiang Wang, Kaikai Meng, Sufang Chen, Liping Zhu, Wenbo Liao

**Affiliations:** ^1^ Department of Horticulture and Landscape Architecture Zhongkai University of Agriculture and Engineering Guangzhou 510225 People's Republic of China; ^2^ State Key Laboratory of Biocontrol School of Life Science Sun Yat‐sen University Guangzhou 510275 People's Republic of China; ^3^ Shenzhen Urban Planning and Land Resource Research Center Shenzhen 518000 People's Republic of China

**Keywords:** genetic diversity, Ixonanthaceae, *Ixonanthes chinensis*, microsatellite marker, transcriptome

## Abstract

**Premise:**

*Ixonanthes* (Ixonanthaceae) consists of between three and 19 species, among which *I. chinensis* and *I. khasiana* are considered vulnerable. Here, 58 microsatellite markers were developed for further conservation of these two *Ixonanthes* species.

**Methods and Results:**

RNA transcripts of *I. chinensis* were sequenced and assembled into 19,545 unigenes, and 994 simple sequence repeat (SSR) loci were identified from 920 contigs. Based on these, 106 primer pairs were designed, 58 were successfully amplified, and 12 demonstrated polymorphism among five populations. The number of alleles per locus varied from three to 10, and the levels of observed and expected heterozygosity ranged from 0.000 to 1.000 and 0.000 to 0.844, respectively. Further assessment of the transferability of the 58 amplifiable primers reported 30 being successfully cross‐amplified in *I. icosandra* and three in *Erythroxylum sinense*.

**Conclusions:**

These novel SSR markers will be useful for future genetic conservation studies on these *Ixonanthes* species.

The genus *Ixonanthes* Jack (Ixonanthaceae) consists of 19 recorded species at present (Kool, 1980). Among them, *I. chinensis* Champ. and *I. khasiana* Hook. f. were assessed as “Vulnerable” in 1998 in the International Union for Conservation of Nature (IUCN) Red List (IUCN, 2018). Further assessment carried out in China has also listed *I. chinensis* in the China Species Red List as “Vulnerable” (Wang and Xie, 2004). However, the classification of *Ixonanthes* species is still controversial. Based on morphological characters, Kool (1980) proposed that (1) the genus *Ixonanthes* should contain only three species: *I. reticulata* Jack, *I. petiolaris* Blume, and *I. icosandra* Jack and (2) *I. chinensis* and *I. khasiana* should be considered as synonyms to *I. reticulata*. These opinions were confirmed by Mabberley (2008).

In China, *I. chinensis* is sometimes harvested for its wood as timber, which is processed for furniture and household products (Zhou and Lin, 2017). Although the survival of this species in the wild is a cause for concern among local researchers and conservationists, no studies have been carried out to assess its genetic diversity. The lack of genetic information on this vulnerable species could be due to the unavailability of useful molecular markers to carry out the work. Therefore, in this study, we have developed useful expressed sequence tag–simple sequence repeat (EST‐SSR) markers for *I. chinensis*. Furthermore, we also examine the cross‐transferability of these markers in the closely related species *I. icosandra* and *Erythroxylum sinense* Y. C. Wu (Erythroxylaceae).

## METHODS AND RESULTS

Total RNA was extracted from fresh leaves of an *I. chinensis* individual (Appendix 1) using a modified cetyltrimethylammonium bromide (CTAB) method (Fu et al., 2005), and a cDNA library was constructed and sequenced using the HiSeq X Ten system (Illumina, San Diego, California, USA). Applying NGS QC Toolkit v2.3.3 (Patel and Jain, 2012), low‐quality reads containing unknown “N” bases or more than 10% bases with a *Q* value less than 20 were removed. Finally, applying Trinity v2.3.2 with default parameters (Grabherr et al., 2011), a total of 21 million high‐quality reads were de novo assembled into 19,545 unigenes with an average length of 517 bp and an N50 length of 621 bp. The raw data and the assembled sequences were deposited in the National Center for Biotechnology Information (NCBI) Sequence Read Archive (SRA) and Transcriptome Shotgun Assembly (TSA) repositories (accession number: SRP127226).

SSRs containing more than six dinucleotide motifs and more than five tri‐, tetra‐, penta‐, and hexanucleotide motifs were searched from the unigenes using the online software MISA (Thiel et al., 2003). A total of 994 SSRs were identified from 920 unigenes, with 32 unigenes containing more than one SSR and 68 unigenes containing compound SSRs. The frequency of EST‐SSRs observed in the *I. chinensis* transcriptome was 4.7%. The most abundant repeat type was trinucleotide (53.8%), followed by dinucleotide (41.2%), tetranucleotide (3.5%), pentanucleotide (0.8%), and hexanucleotide (0.7%) repeat units. A total of 106 primer pairs were successfully designed for these SSR regions using Primer3 (Rozen and Skaletsky, 1999), specifying for an expected PCR product between 100 and 280 bp and an annealing temperature of 55°C.

Fresh leaves were collected from five populations of *I. chinensis* (*n* = 97), one population of *I. icosandra* (*n* = 5), and one population of *E. sinensis* (*n* = 14) (Appendix 1), then dried with silica gel at room temperature. In the first PCR trial, three individuals were randomly selected from each of the five *I. chinensis* populations and used as templates to test the 106 developed primers. DNA extractions and PCR amplifications were performed according to Fan et al. (2013). Among all tested primers, only 58 primers produced distinct bands within the expected size range (Appendix 2), and were thus included in the subsequent analysis. PCR products were then loaded onto a Fragment Analyzer Automated CE System (Advanced Analytical Technologies [AATI], Ames, Iowa, USA) using the QuantiT PicoGreen dsDNA Reagent Kit (Invitrogen, Carlsbad, California, USA). Allele sizes were determined using PROSize version 2.0 software (AATI), which resulted in only 12 primer pairs being polymorphic across the 15 individuals (Table 1).

**Table 1:** Characteristics of 12 polymorphic simple sequence repeat markers isolated from *Ixonanthes chinensis*

Locus	Primer sequences (5′–3′)	Repeat motif	Expected allele size (bp)	Observed size range (bp)	*A*	*T*_a_ (°C)	Putative function [organism]	*E*‐value	GenBank accession no.
LC6	F: AAGATTGCCTTCAACCAGTA	(ATGAG)_5_	333	313–343	7	52	Glutamine‐fructose‐6‐phosphate aminotransferase [isomerizing] 2 [*Carica papaya*]	5e‐22	KX882016
	R: GGACCACGATTACATACAGT								
LC7	F: GGTGAGCCAAGACAAGTG	(CCAAT)_5_	254	254–264	3	58	PREDICTED: uncharacterized protein LOC18587496 [*Theobroma cacao*]	1e‐97	KX882017
	R: GCATTAAGCGTAAGCAACA								
LC12	F: CATTCCACTCCACTCCAAT	(ATGT)_6_	124	120–136	5	55	1,4‐dihydroxy‐2‐naphthoyl‐CoA thioesterase 1 isoform X1 [*Hevea brasiliensis*]	3e‐07	KX882018
	R: GCTGCTGGCTAATTGAGA								
LC22	F: CTCACCATCCTCGCATAC	(TGC)_7_	309	297–315	7	55	PREDICTED: BRI1 kinase inhibitor 1‐like [*Populus euphratica*]	5e‐81	KX882019
	R: CTCTCCTCGTTCCTCCAT								
LC26	F: CTCAGGAGTCAAGCCATC	(CTC)_7_	235	388–415	10	55	Putative SERF‐like protein [*Arachis duranensis*]	2e‐06	KX882020
	R: CTGGACCGTCTCTACCTT								
LC33	F: CGCCATTGTTAGAGAAGGA	(GAA)_7_	175	160–172	5	55	PREDICTED: uncharacterized protein LOC8286849 [*Ricinus communis*]	3e‐10	KX882021
	R: TCACCACTCATCAAGAACC								
LC56	F: TAGCAGCGAAGGAAGAGA	(AAGC)_5_	180	160–176	5	55	—	—	KX882022
	R: GATAGATAGATGGTGAACAAGG								
LC60	F: ACATCGGTAGCAGCATATAG	(TAT)_6_	144	134–155	6	55	PREDICTED: CDPK‐related kinase 4 isoform X2 [*Ricinus communis*]	3e‐28	KX882023
	R: CTAATCACATCTCCTCAACAAG								
LC69	F: TCTTCATGCCAACACTCAG	(TGT)_6_	126	120–135	6	58	—	—	KX882024
	R: ATCACAGCCTCCATCTCC								
LC81	F: CTTGTACTGATCGTTGTTGT	(TGA)_6_	231	231–243	5	55	PREDICTED: uncharacterized protein LOC107428075 [*Ziziphus jujuba*]	4e‐19	KX882025
	R: GCGGAAGCATTCGTATTC								
LC87	F: AGAATACCTGCCAACAATCA	(TAA)_6_	339	339–351	8	58	PREDICTED: probable adenylate kinase 6, chloroplastic [*Ricinus communis*]	6e‐140	KX882026
	R: CGCACTGAACCTTGAAGA								
LC103	F: TCAAGGAATCATCAGAGCAT	(AG)_9_	196	182–188	3	55	Uncharacterized protein LOC110666353 [*Hevea brasiliensis*]	3e‐28	KX882027
	R: AGTGGAGGAGAAGAACAATG								

Note

*A* = number of alleles; *T*_a_ = annealing temperature.

For these 12 primer pairs, PCR amplifications were performed across all 97 individuals from the five natural populations of *I. chinensis*, and their PCR products were first electrophoresed on 10% polyacrylamide denaturing gel and then inspected with the Fragment Analyzer Automated CE System. Scoring errors and null alleles were detected using MICRO‐CHECKER (van Oosterhout et al., 2004); Hardy–Weinberg equilibrium, linkage disequilibrium, the average number of alleles per locus, and the levels of observed and expected heterozygosity were calculated using GenAlEx version 6.5 (Peakall and Smouse, 2012). Results showed that the number of alleles per locus ranged from three to 10 (Table 1), observed heterozygosity ranged from 0.000 to 1.000, and expected heterozygosity ranged from 0.000 to 0.844 (Table 2). Of the 12 polymorphic markers, eight showed significant deviation in Hardy–Weinberg equilibrium for the SZ and YC populations, seven for the HN population, six for the XG population, and five for the HSD population. No significant linkage equilibrium (*P* < 0.05) was detected between locus pairs (Table 2). Further cross‐species amplification was carried out using the initial 58 primer sets on two closely related species, *I. icosandra* (*n* = 5) and *E. sinense* (*n* = 14), and resulted in successful cross‐amplification of 30 primer sets in *I. icosandra* and three in *E. sinense* (Table 3). Our results showed that cross‐transferability of these EST‐SSR markers derived from *I. chinensis* displayed high transferability within other *Ixonanthes* species, but displayed rather low transferability in species of a different genus.

**Table 2:** Genetic diversity of 12 polymorphic SSRs developed for *Ixonanthes chinensis* among five populations.^a^

Locus	HN (*n* = 20)			HSD (*n* = 18)			SZ (*n* = 22)			XG (*n* = 16)			YC (*n* = 21)		
	*A*	*H*_o_ b	*H*_e_	*A*	*H*_o_ b	*H*_e_	*A*	*H*_o_ b	*H*_e_	*A*	*H*_o_ b	*H*_e_	*A*	*H*_o_ b	*H*_e_
LC6	5	0.158***	0.738	5	0.111***	0.673	3	0.045***	0.476	1	0.000*	0.117	6	0.095***	0.642
LC7	3	0.500	0.655	3	0.333**	0.648	3	0.682	0.594	3	0.533	0.638	3	0.524*	0.659
LC12	4	0.950	0.634	2	0.500	0.498	3	0.818	0.648	4	0.813	0.572	4	0.571	0.529
LC22	6	0.550***	0.761	5	0.000	0.000	6	0.409***	0.759	1	0.438***	0.756	6	0.190***	0.630
LC26	6	0.000***	0.770	6	0.176***	0.649	7	0.273***	0.803	5	0.063***	0.701	6	0.095***	0.785
LC33	4	0.350***	0.666	4	0.222***	0.718	4	0.500	0.515	4	0.250***	0.652	5	0.143***	0.704
LC56	4	0.450	0.585	3	0.556	0.573	4	0.364***	0.636	4	0.563	0.615	5	0.476***	0.685
LC60	6	0.950	0.750	4	1.000	0.596	4	1.000***	0.758	5	0.875	0.844	6	1.000	0.693
LC69	5	0.500	0.625	2	0.222	0.198	3	0.455	0.395	2	0.313	0.447	3	0.286	0.357
LC81	3	0.050***	0.524	1	0.000	0.000	4	0.136***	0.520	3	0.188*	0.439	4	0.286***	0.508
LC87	4	0.000***	0.590	4	0.000***	0.599	5	0.273***	0.633	4	0.125***	0.609	5	0.048***	0.756
LC103	3	0.150**	0.476	1	0.000	0.000	2	0.000*	0.087	2	0.063	0.061	1	0.000	0.000

Note

— = not amplified; *A* = number of alleles; *H*_e_ = expected heterozygosity; *H*_o_ = observed heterozygosity; HWE = Hardy–Weinberg equilibrium probabilities; *n* = number of individuals sampled.

^a^Locality and voucher information are provided in Appendix 1.

^b^Deviations from HWE were statistically significant at *P < 0.05, **P < 0.01, and ***P ≤ 0.001.

**Table 3:** Cross‐amplification of 58 microsatellite loci developed for *Ixonanthes chinensis* in *I. icosandra* and *Erythroxylum sinense*.^a^

Locus	*Ixonanthes icosandra*		*Erythroxylum sinense* b	
	Length (bp)	Temperature (°C)	Length (bp)	Temperature (°C)
LC3	—	—	—	—
LC6	—	—	—	—
LC7	—	—	—	—
LC11	250–300	55	—	—
LC12	100–150	55	100–150	55
LC15	150–200	55	—	—
LC16	250–300	61	—	—
LC17	250–300	55	—	—
LC18	—	—	—	—
LC20	200–250	55	—	—
LC22	250–300	55	—	—
LC24	—	—	—	—
LC25	200–300	55	—	—
LC26	250–300	55	—	—
LC29	—	—	—	—
LC30	—	—	—	—
LC33	—	—	—	—
LC36	250–300	55	—	—
LC39	200–250	61	—	—
LC42	250–300	61	—	—
LC45	100–150	61	—	—
LC47	100–150	61	—	—
LC48	—	—	—	—
LC49	200–250	61	—	—
LC51	200–250	61	—	—
LC53	—	—	—	—
LC56	200–250	55	—	—
LC58	200–250	61	—	—
LC59	—	—	—	—
LC60	200	55	—	—
LC61	—	—	—	—
LC65	—	—	—	—
LC66	—	—	—	—
LC67	—	—	—	—
LC69	—	—	—	—
LC70	200–250	61	—	—
LC71	—	—	—	—
LC72	200–250	61	—	—
LC76	250–300	61	—	—
LC78	—	—	—	—
LC79	—	—	—	—
LC81	200–250	55	—	—
LC83	250–300	55	—	—
LC85	—	—	—	—
LC87	250–300	55	—	—
LC89	250–300	55	—	—
LC93	—	—	—	—
LC96	250–300	55	—	—
LC97	—	—	—	—
LC98	—	—	—	—
LC99	200–250	55	—	—
LC100	250–300	55	—	—
LC101	—	—	—	—
LC102	—	—	—	—
LC103	200–250	55	200–250	58
LC104	—	—	>500	48
LC105	—	—	—	—
LC106	—	—	—	—

a Locality and voucher information are provided in Appendix 1.

b The amplifications of *Erythroxylum sinense* were performed in 2× Taq PCR Master Mix (KT201). The lengths were all calibrated by a 50‐bp DNA Ladder (MD108) (Tiangen Biotech Co., Beijing, China).

## CONCLUSIONS

In this study, the transcriptome of *I. chinensis* was established using a de novo sequencing technique. By using this transcriptome library, a total of 58 EST‐SSR markers were developed, of which 12 were polymorphic across the five *I. chinensis* populations. Cross‐amplification of these EST‐SSR markers was also demonstrated on the closely related species *I. icosandra* and *E. sinense*. Through these efforts, our aim to provide ample population genetic information for *Ixonanthes* species was achieved, and the resulting markers could be useful for future studies on species delimitation, taxonomic revision, and genetic conservation of *Ixonanthes* species.

## Data Availability

Microsatellites and raw sequence data for the developed primers have been deposited to the National Center for Biotechnology Information (NCBI). The GenBank accession numbers for the microsatellites are provided in Table 1; the raw sequence data are available in the NCBI Sequence Read Archive (SRA) and Transcriptome Shotgun Assembly (TSA) databases (accession number: SRP127226).

## APPENDIX 1 Voucher and locality information for populations used in this study. Specimens are deposited at the Herbarium of Sun Yat‐sen University (SYS), China.

**Table:**

Species	Population code	Voucher no.	Collection locality	Geographic coordinates	*n*
*Ixonanthes chinensis* Champ.	SZ	*Fan and Guo 1610*	DizhiPark, Shenzhen, Guangdong	22°31′47.44″N, 114°32′10.61″E	22
	HN	*Fan and Guo 210*	Baishuiling, Hainan	18°41′04.08″N, 109°51′11.97″E	20
	HSD	*Fan and Guo 1621*	Heishiding, Guangdong	22°24′12.62″N, 111°30′38.26″E	18
	YC	*Fan and Guo 212*	Yangchun, Guangdong	22°10′23.18″N, 111°47′11.00″E	21
	XG	*Liao and Sun 002*	Hong Kong	22°25′17.63″N, 113°56′14.36″E	16
*Ixonanthes icosandra* Jack	Malaysia	*Fan and Zhao HTBP‐5321*	Gunung Tahan, Pahang state	4°38′30.84″N, 102°9′34.02″E	5
*Erythroxylum sinense* Y. C. Wu	JGS	*Guo and Zhao JGS‐3437*	Jingangshan, Jiangxi	26°33′47.12″N, 114°08′10.20″E	14

Note

*n* = number of individuals sampled.

## APPENDIX 2 Characteristics of 58 microsatellite loci for *Ixonanthes chinensis*.

**Table:**

Locus	Primer sequences (5′–3′)	Repeat motif	Expected allele size (bp)	*T*_a_ (°C)
LC3	F: TCTTACGAGCACGGACTT	(GCA)_8_	257	55
	R: ACCAACAGCAGCATAACAT			
LC6	F: AAGATTGCCTTCAACCAGTA	(ATGAG)_5_	333	52
	R: GGACCACGATTACATACAGT			
LC7	F: GGTGAGCCAAGACAAGTG	(CCAAT)_5_	254	58
	R: GCATTAAGCGTAAGCAACA			
LC11	F: GGTGACTCCAGATATTGATTC	(GGAT)_5_	260	55
	R: AATGACACTTCCGCTATACT			
LC12	F: CATTCCACTCCACTCCAAT	(ATGT)_6_	124	55
	R: GCTGCTGGCTAATTGAGA			
LC15	F: ATTAGTGTAGAGCGAAGTGA	(AGG)_7_	177	55
	R: CTGAACCTGAATCATCTCCT			
LC16	F: TCGGCAAGATAGGAATGTAT	(GCT)_7_	271	61
	R: AGCAGAGGTTCAGAAGGA			
LC17	F: GATAGCCTGGGTAACAATGA	(AGC)_7_	262	55
	R: TTCGGTGGACACAACTCT			
LC18	F: CGTAGACCTGGCAATGTAA	(GCC)_7_	334	55
	R: GGTGGATACTATGCTTGTTG			
LC20	F: ACTGCTCTGGTTCTTCTTC	(GAT)_7_	218	55
	R: AGTGGCTCTATCCTATTCCT			
LC22	F: CTCACCATCCTCGCATAC	(TGC)_7_	309	55
	R: CTCTCCTCGTTCCTCCAT			
LC24	F: CAATGAACAGAAGCACAGAT	(GCT)_6_	298	55
	R: TAGCCAGCGAGAAGAAGA			
LC25	F: ACACAACATCGTCCATCAT	(AGG)_6_	236	55
	R: ACAGCACAAGAAGACAGAG			
LC26	F: CTCAGGAGTCAAGCCATC	(CTC)_7_	235	55
	R: CTGGACCGTCTCTACCTT			
LC29	F: TGACCGATACCAGAGCAT	(GGT)_6_	309	55
	R: AGCATCTTCTTCTTCTTCCA			
LC30	F: CACTTCTTGCTTCTGTTACC	(GGA)_7_	341	55
	R: CGTTGTTGCTGTCTTGTAG			
LC33	F: CGCCATTGTTAGAGAAGGA	(GAA)_7_	175	55
	R: TCACCACTCATCAAGAACC			
LC36	F: CTCCTCGTCGTCCTCTAA	(TAG)_6_	339	55
	R: GTCACCACTAGCATCCTATT			
LC39	F: AAGTGGTGAGAATTGAAGGT	(CTG)_6_	213	61
	R: GCAGAAGTTCGTGTGGAG			
LC42	F: TCCTCAAGCGAGAGTTCT	(GGT)_7_	259	61
	R: CTGTTACTGACTTACTGTTACC			
LC45	F: CCTGGTCGGTCACATAGA	(GGC)_7_	155	61
	R: CGCTCCTTCTCATCATCTC			
LC47	F: TTGCCGCTCTTTACATTTG	(GATG)_6_	157	61
	R: AACGAAGGAAGACCAACAG			
LC48	F: CTGTTCCACCTTCACTGA	(TCTT)_5_	219	55
	R: CGTATGAATGGAGAGTAAGAG			
LC49	F: TTCAATCCGAGTAATGATGG	(GCA)_7_	241	61
	R: CGCTCCACTTCCTAATGA			
LC51	F: CATCCGCCGAATAATGAAC	(CTT)_6_	244	61
	R: GATTGTTGTCTCGCTTCTT			
LC53	F: TTCTCCTCCAGTTCTCCAT	(ATAC)_5_	122	55
	R: AACACTCCAGAGCCAGAG			
LC56	F: TAGCAGCGAAGGAAGAGA	(AAGC)_5_	180	55
	R: GATAGATAGATGGTGAACAAGG			
LC58	F: TTCACATCACAGGTACAGAT	(AGAT)_5_	212	61
	R: GCCAGAAGAGGAGGTATTG			
LC59	F: TGCGTTCGGTAATGACTTA	(CAT)_5_	258	55
	R: TCAGAATCAAGCCAGGATG			
LC60	F: ACATCGGTAGCAGCATATAG	(TAT)_6_	144	55
	R: CTAATCACATCTCCTCAACAAG			
LC61	F: GCCAACAACAACAACCATT	(GCT)_6_	216	55
	R: CGTCAGCCATAGTGTCATAA			
LC65	F: CTCTGATACTGTCCACTTCC	(CAG)_5_	258	55
	R: TGTTCGTTCCTCCATTCTC			
LC66	F: AGAAGAGGAAGAAGAGAAGGT	(GGT)_6_	130	55
	R: CGTCGTCGTTGCTGTTAG			
LC67	F: ATGCGAAGGTGAGTCAAC	(TA)_9_	279	55
	R: TACAGATGAGTCGTAAGAAGG			
LC69	F: TCTTCATGCCAACACTCAG	(TGT)_6_	126	58
	R: ATCACAGCCTCCATCTCC			
LC70	F: GTAAGGGCTAAGACCAGAAA	(CAT)_6_	216	61
	R: ACCTCCAAGCACATCCAT			
LC71	F: TCGTCCTTCTCCTTAACTTC	(TCT)_6_	205	61
	R: TGCTGTTGCTTCACTTCA			
LC72	F: TCTGAACTCGCTTTCCATC	(TTC)_6_	228	61
	R: AACACGCTTATCAACAACAC			
LC76	F: TTGTGTATGACGGCTCTG	(AAG)_6_	278	61
	R: AGGTGGAAGACAAGTATTCA			
LC78	F: AGGTTCTGCCAATAATGTCA	(AAC)_6_	349	55
	R: GCTGTTGTTATTCTGGATGT			
LC79	F: TGCCTCACTTGTTCTTCTC	(CCT)_5_	285	55
	R: ATCATCAGCGTCTCCAATC			
LC81	F: CTTGTACTGATCGTTGTTGT	(TGA)_6_	231	55
	R: GCGGAAGCATTCGTATTC			
LC83	F: AGCACAATCCTCCTCGTA	(AGC)_6_	348	55
	R: CTCCTCTTGTTCTCCTCAG			
LC85	F: AAGAACAACAAGAGGATGC	(CAC)_6_	276	55
	R: GCGTCCGTAATCATAAGC			
LC87	F: AGAATACCTGCCAACAATCA	(TAA)_6_	339	58
	R: CGCACTGAACCTTGAAGA			
LC89	F: CTCAATCAAGATACGGTTGT	(GTG)_6_	252	55
	R: GAGACGGAATTGTTCATAGG			
LC93	F: GCCAATCCAACACAATGC	(TAA)_6_	163	55
	R: CGGTGCTCATATCTCTTCC			
LC96	F: GGATTCCAAGTGCTTAACAT	(CCA)_6_	293	55
	R: GAAGACAAGGCGGTAGAA			
LC97	F: GGAATGCCACAGAACAAC	(CAC)_6_	303	55
	R: ATGCTCAATGTACTCTCCTC			
LC98	F: AATGGCTGGCAATGAGAA	(TAA)_5_	342	55
	R: GCGGTATCTTCCAACACT			
LC99	F: AGCCTTCTTCTCCTCTTCA	(CAT)_5_	149	55
	R: GGTCTGGTGTCACTGTTG			
LC100	F: AATCGCATAGTCGCAAGG	(GAT)_6_	256	55
	R: AAGGCAAGCACATCAAGT			
LC101	F: GTTAAGGTGGAGCAGGAG	(AGC)_5_	216	55
	R: TAGCGGATGGTTCTTCTTC			
LC102	F: TTCGCTGGTTGTCAAGTT	(CAT)_6_	201	55
	R: CTCATATCGGTTCCAATCG			
LC103	F: TCAAGGAATCATCAGAGCAT	(AG)_9_	196	55
	R: AGTGGAGGAGAAGAACAATG			
LC104	F: TCTCCTCTTCTCCTCTTCTT	(CTT)_6_	252	55
	R: AACCTAGCAACACCTCCT			
LC105	F: TGGAAGGAATCTGTCACTAC	(GAT)_6_	207	55
	R: CTGATGGATCGACCGTAAT			
LC106	F: GTATGCTAGTGGTCACCTAC	(ACC)_6_	115	55
	R: GCTATGTTGTCGGCTTCC			

Note

*T*_a_ = annealing temperature.
